# Site downsizing: a blessing or a curse?

**DOI:** 10.1192/bjo.2021.605

**Published:** 2021-06-18

**Authors:** Benjamin Waterhouse, Ben Attwood

**Affiliations:** Camden & Islington NHS Trust

## Abstract

**Aims:**

To measure staff wellbeing and morale, which in 2015 was described by CQC as 'low', following a downsizing of premises.

**Background:**

In 2019, due to loss of mental health funding, Camden & Islington NHS Foundation Trust controversially sold the much-loved Queen Anne-style mansion Lyndhurts Gardens. The Rehab & Recovery team caring for those with serious mental illness were relocated to one floor of the much smaller Daleham Gardens. It was hypothesed by the authors that this would impact negatively on the already unhappy workforce.

**Method:**

The same staff wellbeing survey was used as in in 2015 (following CQC's description of 'poor' morale), nearly 5 years on and following the site relocation. All clinical, managerial and administrative staff members were encouraged to participate by posting their survey anonymously in a make-shift postbox. Sweet treats were used to encourage participation within the busy team.

**Result:**

Qualitative and quantitative data were collected from the team (response rate 44%). Exact tables will be shown but in summary; in the new building there was an increase in the number of staff who felt they could use initiative at work, make improvements at work, looked forward to going into work and felt enthusiastic about their job. Improvement cited were 'increased socials after work' and consequent 'wellbeing', in addition to 'good team atmosphere', 'good team work' and 'good relationships' in the new space. Further ideas were generated for additional improvements going forward.

**Conclusion:**

Whilst caseloads and workloads are often cited as the cause of burnout, and indeed other changes in the 5 years could act as confounders, the design of work buildings is not to be underestimated. Contrary to what the authors had suspected, 'bigger' was not necessarily 'better' and a more condensed working environment made for greater togetherness amongst the team. In this age where economically desperate NHS trusts are forced to sell off their prized assets, observations that this is not always at the detriment of staff morale will provide some cause for optimism.

